# Selective estrogen receptor modulators regulate reactive microglia after penetrating brain injury

**DOI:** 10.3389/fnagi.2014.00132

**Published:** 2014-06-20

**Authors:** George E. Barreto, Maria Santos-Galindo, Luis Miguel Garcia-Segura

**Affiliations:** ^1^Departamento de Nutrición y Bioquímica, Facultad de Ciencias, Pontificia Universidad JaverianaBogotá, D.C., Colombia; ^2^Instituto Cajal, Consejo Superior de Investigaciones CientificasMadrid, Spain

**Keywords:** brain injury, raloxifene, tamoxifen, microglia, neuroprotection

## Abstract

Following brain injury, microglia assume a reactive-like state and secrete pro-inflammatory molecules that can potentiate damage. A therapeutic strategy that may limit microgliosis is of potential interest. In this context, selective estrogen receptor modulators, such as raloxifene and tamoxifen, are known to reduce microglia activation induced by neuroinflammatory stimuli in young animals. In the present study, we have assessed whether raloxifene and tamoxifen are able to affect microglia activation after brain injury in young and aged animals in time points relevant to clinics, which is hours after brain trauma. Volume fraction of MHC-II^+^ microglia was estimated according to the point-counting method of Weibel within a distance of 350 μm from the lateral border of the wound, and cellular morphology was measured by fractal analysis. Two groups of animals were studied: (1) young rats, ovariectomized at 2 months of age; and (2) aged rats, ovariectomized at 18 months of age. Fifteen days after ovariectomy animals received a stab wound brain injury and the treatment with estrogenic compounds. Our findings indicate that raloxifene and tamoxifen reduced microglia activation in both young and aged animals. Although the volume fraction of reactive microglia was found lower in aged animals, this was accompanied by important changes in cell morphology, where aged microglia assume a bushier and hyperplasic aspect when compared to young microglia. These data suggest that early regulation of microglia activation provides a mechanism by which selective estrogen receptors modulators (SERMs) may exert a neuroprotective effect in the setting of a brain trauma.

## Introduction

Microglia are considered as the macrophages of the brain. Trauma to the brain causes increased production of reactive oxygen species and oxidative stress, neuronal damage (Barreto et al., 2011) and elicits activation of astrocytes and microglia. Microglia respond to brain insults by activation, assuming a reactive state, which involves increased production of pro-inflammatory molecules, including chemokines, cytokines, adhesion molecules, metalloproteinases, hyperplasia of cell body and hypertrophy of cellular processes (Aloisi, 2001; Loane and Byrnes, 2010; Xiong et al., 2011; Hernandez-Ontiveros et al., 2013). Increased on site inflammation may prevent tissue recover and repair (Adelson et al., 2012), and a therapeutic strategy that may limit the release of molecules that potentiate damage is of potential interest.

Estrogens may exert neuroprotection after brain injury by regulating reactive astrocytes and microglia (Garcia-Estrada et al., 1993; García-Estrada et al., 1999; Barreto et al., 2007, 2009). Despite these protective effects of estradiol on brain, this hormone may produce some side effects in peripheral tissues, especially in women submitted to post-menopausal hormonal therapy. The transcriptional activity of estrogen receptors (ER) is regulated by their association with transcriptional cofactors that have a tissue or cell specific expression (Klinge, 2000; Belandia and Parker, 2003). Therefore, in this context, several estrogen receptor ligands, known as selective estrogen receptors modulators (SERMs), are able to exert tissue specific actions, acting as ER agonists in some tissues and as ER antagonists in others. Although the effect of estradiol on brain after damage is well explored, the role that SERMs may have is not clear.

Previous studies showed that raloxifene and tamoxifen, two known SERMs used in clinics, reduce microglial activation induced by lipopolysaccharide (Suuronen et al., 2005; Tapia-Gonzalez et al., 2008), irradiaton-induced brain injury (Liu et al., 2010), and spinal cord injury (Ismailoğlu et al., 2010, 2013). Although these studies demonstrate a potential effect of SERMs on the inflammation resolution following different brain insults, all of them focused on young subjects. Although one study has demonstrated that raloxifene reduces the number of microglia cells in the hippocampus of female mice during aging (Lei et al., 2003), it is unknown if SERMs maintain their potency to decrease reactive microgliosis after brain injury in aged animals. Therefore in this study we have compared the effects of raloxifene and tamoxifen on reactive microgliosis after brain injury in young and aged female rats.

## Materials and methods

### Animals and experimental treatments

Wistar albino female rats from the Complutense University animal colony (Madrid, Spain) were kept on a 12:12 h light/dark schedule and received food and water *ad-libitum*. Animals were handled in accordance with the guidelines published in the NIH Guide for the Care and Use of Laboratory Animals, the principles presented in the Guidelines for the Use of Animals in Neuroscience Research by the Society for Neuroscience and following the Spanish Royal Decree 53/2013 about protection of experimental animals, in close agreement with the European Communities Council Directives 86/609/EEC and 2010/63/UE. Experimental procedures were approved by our institutional animal use and care committee. Special care was taken to minimize suffering and to reduce the number of animals used to the minimum required for statistical accuracy.

### Experimental design

In this study we have assessed the effect of estradiol, raloxifene and tamoxifen on reactive microglia in ovariectomized rats, a model that imitates menopause. To determine whether the effects of estrogenic compounds are altered by aging, we studied two groups of animals: (1) young rats ovariectomized at 2 months of age; and (2) aged rats castrated at 18 months of age. All animals received brain injury 15 days after ovariectomy.

### Brain injury

Animals were bilaterally ovariectomized under halothane anesthesia (Fluothane, AstraZeneca Farmacéutica, Madrid, Spain). For brain surgery, animals were anesthetized with halothane and placed in a stereotaxic apparatus (David Kopf Instruments, Tujunga, CA, USA). An incision of the scalp was made and the cranium exposed. Then, a unilateral opening of the skull was made with a dental drill. A solid stainless steel cannula, with a 0.45 mm outer diameter, was used to make a longitudinal stab wound in the left hemisphere. The cannula was positioned at 2 mm lateral to the midline in young rats, at 2.4 mm lateral to the midline in aged rats and at 2 mm posterior to bregma in both age groups and introduced into the brain until the tip reached a depth of 5.5 mm. Then, the cannula was displaced caudally 3 mm (bregma: −5 mm) and finally removed from the brain. Bleeding was inhibited by compression with a gel-foam sponge. The scalp wound was sutured with surgical silk.

### Treatments with estrogenic compounds

Animals received one subcutaneous injection of 17β-estradiol (E2758, Sigma-Aldrich, St. Louis, Mo; 100 μg/Kg), raloxifene (R1402, Sigma-Aldrich, 1 mg/Kg), and tamoxifen (T5648, Sigma-Aldrich, 1 mg/Kg) after injury, a second injection 24 h later and a third injection 48 h later. Thus, the estrogenic compounds were administered during the period of glial activation, and doses of estradiol and estrogenic compounds were selected on the basis of previous studies. This dose of estradiol is known to stimulate neuroprotective signaling cascades and to exert neuroprotection in the hippocampus (Barreto et al., 2009). These effects may be at least in part due to the high levels of the hormone achieved shortly following the injections. The doses of raloxifene and tamoxifen were previously shown to be neuroprotective in a model of brain trauma *in vivo* and to reduce microglial activation in a model of brain inflammation (Tapia-Gonzalez et al., 2008; Barreto et al., 2009).

### Tissue fixation and immunohistochemistry

One week after brain injury, animals were deeply anesthetized with pentobarbital (100 mg/kg, Normon Veterinary Division, Madrid, Spain) and perfused through the left cardiac ventricle, first with 50 ml saline solution (0.9% NaCl) and then with 250 ml fixative solution (4% paraformaldehyde in 0.1 M phosphate buffer, pH 7.4). Brains were removed and immersed overnight at 4°C in the same fixative solution and then rinsed with phosphate buffer. Coronal sections of the brain, 50 μm thick, were obtained using a Vibratome (VT 1000 S, Leica Microsystems, Wetzlar, Germany).

Immunohistochemistry was carried out on free-floating sections under moderate shaking. All washes and incubations were done in 0.1 M phosphate buffer pH 7.4, containing 0.3% bovine serum albumin and 0.3% triton X-100. The endogenous peroxidase activity was quenched for 10 min at room temperature in a solution of 3% hydrogen peroxide in 30% methanol. After several washes in buffer, sections were incubated overnight at 4°C with a mouse monoclonal antibody for the Mayor Histocompatibility Complex type II (MHC-II; MRC-OX6, MCA46G, Serotec, Bicester, UK; diluted 1:300), a marker of reactive microglia. Sections were then rinsed in buffer and incubated for 2 h at room temperature with biotinylated goat anti-mouse immunoglobulin G (diluted 1:300; Pierce, Rockford, IL, USA). After several washes in buffer, sections were incubated for 90 min at room temperature with avidin-biotin-peroxidase complex (diluted 1:250; ImmunoPure ABC peroxidase staining kit, Pierce). The reaction product was revealed by incubating the sections with 2 μg/ml 3,3′-diaminobenzidine (Sigma-Aldrich) and 0.01% hydrogen peroxide in 0.1M phosphate buffer. Some sections were counterstained with toluidine blue. Then, sections were dehydrated, mounted on gelatinized slides, coverslipped and examined with a Leica DMRB-E microscope.

### Morphometric analysis

Only brains that showed a complete lesion from the dorsal to the ventral limit of the dorsal hippocampus were selected for morphometry. The high density of MHC-II immunoreactive cell bodies and processes in the proximity of the wound impeded the accurate identification of individual cells. Therefore, the volume fraction of MHC-II immunoreactive microglia was estimated according to the point-counting method of Weibel (1979). The outline of the hippocampus lateral to the border of the wound and the areas filled with immunoreactive material were drawn on a paper using a Leitz microscope equipped with a camera lucida. A transparent point grid was superimposed on the drawings. The total number of points falling on the hippocampus (reference volume) and the number of points falling on immunoreactive material were counted within a distance of 350 μm from the lateral border of the wound. The area associated to each point was 614 μm^2^. The volume fraction of immunoreactive material was calculated for each animal as the ratio of the sum of the number of points falling on immunoreactive material vs. the sum of the number of points falling in the reference volume. At least three sections were evaluated for each animal. All counts were performed on coded sections and drawings.

### Morphological assessment

#### Preselection of reactive microglia

The MHC-II^+^ microglia examined in this section were located in the stratum radiatum at a distance of 100–200 μm from the wound. Only cells with visible nuclei and complete processes not damaged by tissue sectioning were selected for analysis. Cells were selected randomly using a scale generated automatically (available on the Internet).^1^

#### Image acquisition

Black and white images of MHC-II^+^ microglia were obtained using a digital camera attached to a Zeiss axiovert inverted fluorescent microscope (Zeiss, Germany). The images were processed using ImageJ software (developed at the USA National Institutes of Health and available on the Internet).^2^ Under a 40X objective, cells were picked randomly in each selected area, and the binary overlay of a cell was created using thresholding procedure. In this method, all pixels with their gray level values higher than the threshold value were treated as belonging to the cell image. Other pixels were treated as background. For each cell the appropriate threshold value was defined manually at the level at which the binary overlay completely covered the whole cell body and processes. Finally, the binary silhouette of the whole cell was reduced to its one-pixel outline for estimation of the fractal dimensions with the FracLac 2.5 ImageJ plug-in (A. Karperien, Charles Stuart University).

### Quantitative fractal analysis

Fractal analysis was carried out on binary images using dilation method. The method, originally devised by Flook (1978), is based on the Minkowski-Bouligand dimension (Mandelbrot). The simple method can be easily assessed by typical image processing software, so is very often used to determine the fractal dimension of cell images. With this method, each pixel in the cell outline was replaced with a disk of a diameter varying from 3–61 pixels. Then the area of the widened outline divided by the diameter of structuring element was plotted against this diameter in a log-log scale. The slope of the regression line (S) is related to the fractal dimension (D) by D = 1 − S. In addition, the following variables were measured in this study: the area of the cell silhouette (the cell area), and the arbor area—the area of the convex polygon obtained by connecting the tips of the longest astrocytic processes (also known as the convex hull area). We measured the solidity factor, obtained by the division of the cell silhouette by the arbor area, and lacunarity values.

### Statistical analysis

The *n* used for statistical analysis was the number of animals (*n* = 4–6). Data were analyzed by one-way analysis of variance (ANOVA) followed by the Tukey *post hoc* test for paired comparisons, with *p* < 0.05 considered to be significant. For comparison of two variables (age and treatment), data were analyzed by two-way ANOVA followed by Bonferroni post-tests to determine the interaction of aging and treatment, or each factor alone.

## Results

### SERMs decrease reactive microglia in young animals

**Figure 1:**
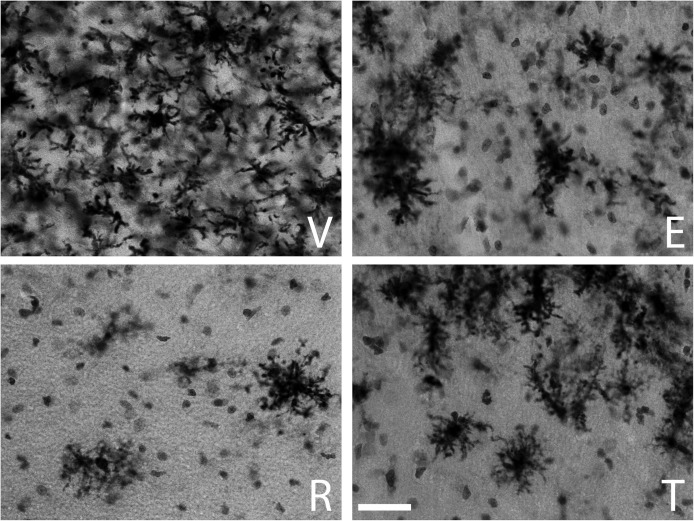
**MHC-II immunoreactive microglia in the CA1 stratum radiatum at a distance of approximately 100–200 µm from the lateral border of the wound in young animals**. The panels illustrate representative examples from ovariectomized young rats after administration of (V) vehicle, (E) estradiol, (R) raloxifene or (T) tamoxifen. All figures are at the same magnification. Scale bar, 50 um.

**Figure 2:**
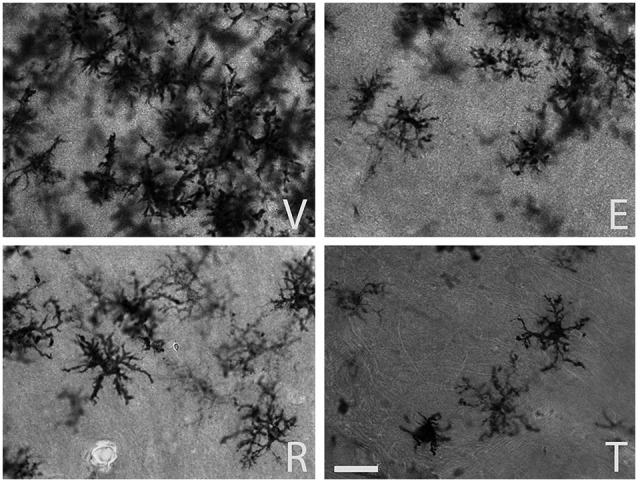
**MHC-II immunoreactive microglia in the CA1 stratum radiatum at a distance of approximately 100–200 µm from the lateral border of the wound in aged animals**. The panels illustrate representative examples from ovariectomized aged rats after administration of (V) vehicle, (E) estradiol, (R) raloxifene or (T) tamoxifen. All figures are at the same magnification. Scale bar, 50 um.

The qualitative inspection of the sections immunostained for MHC-II to detect reactive microglia revealed a prominent microgliosis along the borders of the wound in both young and aged animals treated with vehicle. However, animals treated with estradiol, raloxifene, tamoxifen showed a lower cellular density compared to vehicle animals (Figures 1 and 2).

**Figure 3:**
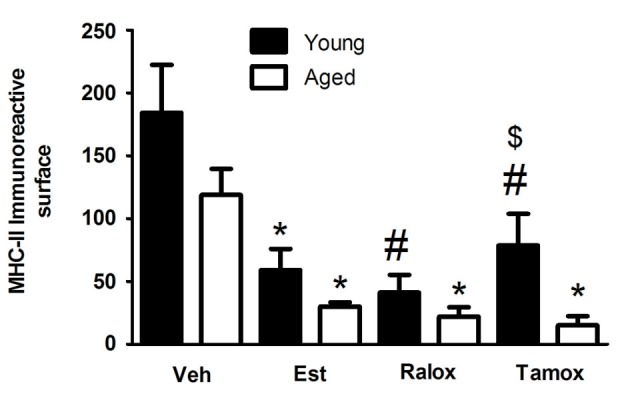
**Volume fraction of MHC-II immunoreactive microglia within a distance of 350 µm from the lateral border of the wound in young and aged animals injected with vehicle (Veh) *n* = 6 (young) and 5 (aged), estradiol (Est) *n* = 4 (young and aged), raloxifene (Ralox) *n* = 6 (young) and 4 (aged), or tamoxifen (Tamox) *n* = 5 (young) and 4 (aged)**. Data are represented as means +/− SEM. * Significant difference (*P* < 0.05) vs. vehicle values. * *P* < 0.05 vs. vehicle (young); # *P* < 0.05 vs. vehicle (aged); $ *P* < 0.05 young vs. aged treated with same SERM.

The morphometric analysis with the Weibel method confirmed the qualitative observations. The volume fraction of MHC-II immunoreactive microglia showed a significant decrease in the young animals that received estradiol (*P* = 0.009), raloxifene (*P* = 0.002) and tamoxifen (*P* = 0.049) in early treatment (Figure 3).

### Effects of aging and SERMs on reactive microglia after injury

To assess the effect of estrogenic compounds on reactive microglia in aged animals following traumatic brain injury, we measured microglia volume fraction and changes in cell morphology by fractal analysis. Our previous observations showed that the age affected the cellular density in animals injected with vehicle (Figure 2), and that the SERMs reduced the fraction of reactive microglia reactive bordering the wound. To support these statements, we assessed the volume fraction of reactive microglia in aged animals after the treatment with estrogenic compounds. Reactive microglia were significantly less numerous in animals treated with estradiol (*P* = 0.03), raloxifene (*P* = 0.02) or tamoxifen (*P* = 0.006) (Figure 3).

### Correlation of microglia reactivity and morphology with aging and treatment

Two-way ANOVA showed no interaction between age and treatment in reactive microglia (*P* = 0.66). On the other hand, each factor alone, age (*F* = 7.136; *P* = 0.0121) or treatment (*F* = 12.65; *P* < 0.0001) affects microglia immunoreactive for MHC-II (Figure 3).

**Figure 4:**
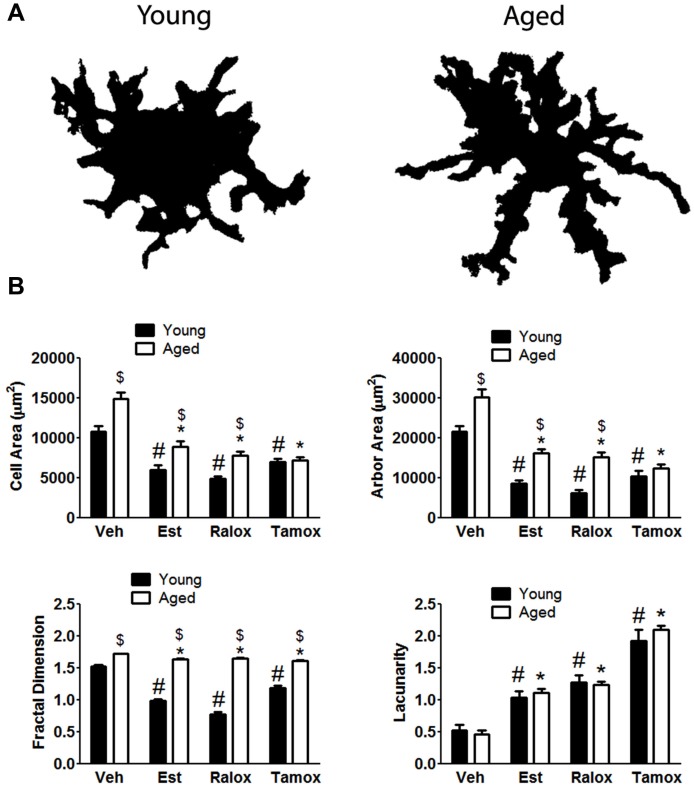
**(A)** The upper panel indicates age differences on microglia morphology with aging in animals treated with vehicle. Fractal analysis shows that microglia reactivity is characterized by changes in both cell body and processes. **(B)** Cell shape is hypertrophied and bushier in control cells (vehicle, V) compared to those cells from animals treated with estradiol (Est), raloxifene (Ralox) or tamoxifen (Tamox). * *P* < 0.05 vs. vehicle (young); # *P* < 0.05 vs. vehicle (aged); $ *P* < 0.05 young vs. aged treated with same SERM.

To quantitatively assess microglia morphology in response to brain injury with aging, we performed fractal analysis (Barreto et al., 2012) of selected individual cells from the same area used for counting. Changes in the complexity of microglia expressing MHC-II were quantified by calculating cell area, arbor area, fractal dimension, and lacunarity values. In the injured brains, MHC-II^+^ microglia assumed a wide spectrum of forms, from small cells with short to large, well-developed cells after penetrating injury in aged animals. Our results show that cell complexity increases with aging (Figure 4A). The cell (*P* < 0.0001) and arbor (*P* < 0.0001) areas were lower in young animals treated with SERMs, in comparison to those in vehicle (Figure 4B), reflecting a decreasing microglia cell body and processes when SERMs treatments are administered. With respect to the fractal dimension (Figure 4B), the mean values characterizing the SERMs post-injury groups were lower (*P* < 0.0001) than those observed in vehicle. We also analyzed lacunarity values, which are sought to be a measure of non-uniformity (heterogeneity) of structure or the degree of structural variance within an object, in this case how complex microglia are. Significant high lacunarity values are found in estradiol (*P* = 0.0002), raloxifene (*P* = 0.0006) or tamoxifen (*P* < 0.0001) treated young animals, in comparison to low values in vehicle (Figure 4B). For cell morphology analysis in aged animals, we observed significant differences in cell and arbor areas (*P* < 0.0001 for both parameters), fractal dimension (*P* < 0.0001) and lacunarity (*P* < 0.0001) in animals treated with SERMs in comparison to vehicle-treated subjects (Figure 4B), suggesting an increased complexity of microglia after injury, and that SERMs were able to reduce the reactive state of these cells.

An effect of treatment was observed in all analyzed parameters: Cell area (*F* = 52.63; *P* < 0.0001), arbor area (*F* = 67.43; *P* < 0.0001), Fractal dimension (*F* = 102.4; *P* < 0.0001) and lacunarity (*F* = 85.52; *P* < 0.0001). On the contrary, age alone only affects cell area (*F* = 36.43; *P* < 0.0001), arbor area (*F* = 58; *P* < 0.0001) and fractal dimension (*F* = 988.3; *P* < 0.0001). Two-way ANOVA showed no significant interaction between aging and treatment only in lacunarity (*P* = 0.61) (Figure 4B).

## Discussion

Microglia become more activated and reactive with aging and assume different morphological states (Peters et al., 1991; Ogura et al., 1994; Sheffield and Berman, 1998; Ye and Johnson, 1999; Kumar et al., 2013) indicating that this altered state may potentiate damage and increase inflammation following brain injury. In the present study we assessed the volume fraction of microglia immunoreactive for MHC-II and analyzed their morphology in young and aged animals subjected to penetrating brain injury. Our findings indicate that SERMs were able to decrease microgliosis, which can be interpreted as the fraction of reactive microglia and the morphological aspect these cells assume bordering the wound. Previous studies have shown that estrogen and the SERM, raloxifene, reduce microglia in aged female mice (Lei et al., 2003), demonstrating a potential neuroprotective action by modulating reactive glia.

The aim of our study was to assess whether SERMs were able to reduce microglia activation in aged females under conditions that imitate menopause. After reproductive aging, rats may enter in constant estrus or constant diestrus. Indeed, Wistar female rats in our colony still maintain relatively high estrogen levels in plasma at 18 months (68 ± 9 pg/ml^−1^). Thus, aged female rats are not a good model for menopause. To imitate menopause is necessary to use ovariectomized rats. We compared aged ovariectomized animals with young ovariectomized animals. Both groups of animals represent models of menopause, but in our experimental design we include the variable age. Interestingly, previous evidences have shown a correlation with aging and/or menopause with increased traumatic brain injury (Onyszchuk et al., 2008; Sandhir et al., 2008; Kumar et al., 2013; Sun et al., 2013), supporting our experimental approach as a reliable model to study the effects of aging and menopause in injured animals.

Previous studies reported the kinetics of microglia activation in aged brain (Kyrkanides et al., 2001; Sandhir et al., 2008), but this is the first study to assess this activated state of microglia in aged vs. young subjects, and propose the SERMs as a potential therapy to control microgliosis, even in animals with advanced aging. Concerning the treatment scheme: (i) we sought to assess SERMs effectiveness in time points relevant to clinics, which is hours after brain trauma; (ii) this period reflects the initial stage of microglia activation, and we hypothesized that raloxifene and tamoxifen regulate the activated state of microglia in the first 48 h following injury; and (iii) the doses of SERMs used in the present have been previously shown to be effective to control astrogliosis following penetrating brain injury (Barreto et al., 2009).

Activation of microglia was reported to be neuroprotective in ischemic models (Lalancette-Hébert et al., 2007), despite its harmful effects to neurons when activation is excessive (Heppner et al., 2005; Marchetti and Abbracchio, 2005). A proper control of microglial reactivity by SERMs may represent a potential therapeutic strategy in brain injuries. The SERMs may have actions on reactive microglia via estrogen receptor (ER) signaling, as these cells express both ERβ (Takahashi et al., 2004) and ERα (Sierra et al., 2008; Tapia-Gonzalez et al., 2008). The expression of the latter may indicate that part of the anti-inflammatory effects of SERMs on microglial cells is sought to happen by activation of this classical estrogen receptor. Interestingly, some unpublished data from our lab demonstrate that reactive microglia is reduced in young animals treated with 2,3-bis(4-hydroxyphenyl)-propionitrile (DPN) (estrogen receptor beta agonist; 93.95 ± 2.41), 4,4′,4″-(4-propyl-[1H]-pyrazole-1,3,5-triyl)trisphenol (PPT) (68.99 ± 6.42) and combined DPN+PPT (52.99 ± 2.23) in comparison to controls (184.28 ± 15.59), suggesting that both receptors may be mediating these protective effects in our model. Besides SERMs effects on microglia activation (Suuronen et al., 2005; Tapia-Gonzalez et al., 2008; Liu et al., 2010; Arevalo et al., 2012), previous studies also showed that raloxifene and tamoxifen induced upregulation of glutamate transporters (Karki et al., 2014), potentiation of mitochondrial superoxide dismutase (Wakade et al., 2008), improves functional outcome following spinal cord injury (Tian et al., 2009), and reduces inflammation via estrogen receptor beta (Baker et al., 2004). Since traumatic brain injury induces a complex cascade of events, which involves increased production of pro-inflammatory molecules, activating microglia, thus augmenting tissue damage and neuronal death, SERMs may act by decreasing the production of inflammatory cytokines secreted by microglia and improve the outcome in both aged and young subjects.

Interestingly, we observed that the volume fraction of MHC-II microglia is lower in aged animals in comparison to young subjects. These observations are supported by other reports, where microglia is found reduced in aged rat brain following excitotoxic damage (Campuzano et al., 2011), intracerebral hemorrhage (Wasserman and Schlichter, 2008) and with normal aging (Cerbai et al., 2012). Although our results indicate that reactive microglia is decreased in aged animals, and that SERMs lowered this number, the reactive-like state of microglia, assessed by fractal analysis, in aged subjects is higher than that from young animals.

Microglia morphology at different states has been widely assessed by fractal analysis (Soltys et al., 2001, 2005; Karperien et al., 2013; Torres-Platas et al., 2014). An important goal in many branches of science, especially in cellular biology and medicine is the quantitative analysis of the structures and their morphology. The morphology can be analyzed in many ways, such as filling cells with Lucifer yellow dye (Wilhelmsson et al., 2006), or in particular by the fractal analysis (Schaffner and Ghesquiere, 2001; Soltys et al., 2001, 2005; Barreto et al., 2012). In the present study, the quantitative analysis showed that the volume fraction of MHC-II-immunoreactive microglia decreased when animals are treated with SERMs. To assess microglia morphology in response to brain injury, we assigned a fractal dimension to randomly picked individual microglia from the same area indicated above. Other parameters, thus, can also be assessed, including cell and arbor areas. We observed that cell and arbor area increased in vehicle-treated young animals, indicating important changes in the cell body and processes. Upon treatment with SERMs, cell complexity is found reduced in young animals. Fractal dimensions provided a measure of how completely an object fills a specified region, in this case how microglia cellular body and processes fill a specific area. Apart from the fractal dimension, an important part of the fractal analysis is the lacunarity measurement which, roughly describing, characterizes the distribution of gaps in the fractal. We showed here that lacunarity values increase in young animals treated with SERMs, indicating “low complexity” of a given object. If we apply these concepts into our study, microglia in animals treated with SERMs have lesser branches and processes, and smaller cell bodies compared to vehicle, which is characterized by larger and increased number of processes and a hypertrophied cellular body.

Effects of aging on microglial morphology upon injury are also noted. Aged reactive microglia assume a bushier and hyperplasic shape when compared to young microglia. Indeed, fractal dimension, a measure of complexity, indicates that aged microglia present significant higher values in comparison to young animals. These observations demonstrate that, although the number of reactive microglia is found reduced in aged animals, microglia in these animals are more reactive, and that SERMs were able to regulate this activated-like state.

## Conflict of interest statement

The authors declare that the research was conducted in the absence of any commercial or financial relationships that could be construed as a potential conflict of interest.

## Footnotes

^1^http://www.random.org/integers

^2^http://rsb.info.nih.gov/ij/
